# Rare codon content affects the solubility of recombinant proteins in a codon bias-adjusted *Escherichia coli *strain

**DOI:** 10.1186/1475-2859-8-41

**Published:** 2009-07-24

**Authors:** Germán L Rosano, Eduardo A Ceccarelli

**Affiliations:** 1 Molecular Biology Division, Instituto de Biología Molecular y Celular de Rosario (IBR), CONICET, Facultad de Ciencias Bioquímicas y Farmacéuticas, Universidad Nacional de Rosario, Suipacha 531. S2002LRK Rosario, Argentina

## Abstract

**Background:**

The expression of heterologous proteins in *Escherichia coli *is strongly affected by codon bias. This phenomenon occurs when the codon usage of the mRNA coding for the foreign protein differs from that of the bacterium. The ribosome pauses upon encountering a rare codon and may detach from the mRNA, thereby the yield of protein expression is reduced. Several bacterial strains have been engineered to overcome this effect. However, the increased rate of translation may lead to protein misfolding and insolubilization. In order to prove this assumption, the solubility of several recombinant proteins from plants was studied in a codon bias-adjusted *E. coli *strain.

**Results:**

The expression of eight plant proteins in *Escherichia coli *BL21(DE3)-pLysS and BL21(DE3)-CodonPlus-pRIL was systematically studied. The CodonPlus strain contains extra copies of the *argU*, *ileY*, and *leuW *tRNA genes, which encode tRNAs that recognize the codons AGA/AGG, AUA and CUA, respectively (RIL codons). The level of expression and solubility of the recombinant proteins were analyzed by means of sodium dodecyl sulfate polyacrylamide gel electrophoresis and Western blotting. We found that for all proteins the solubility was at least 25% in the BL21(DE3)-pLysS strain. However, when expressed in the BL21(DE3)-CodonPlus-pRIL strain, proteins having more than 5% of amino acids coded by RIL codons were localized mainly in the insoluble fraction. Also, their expression caused retarded growth and low cell yield in the codon bias-adjusted strain at all temperatures tested. On the contrary, the solubility of proteins containing less than 5% of amino acids coded by RIL codons remained unchanged in both strains and their expression caused no effect on cell growth.

**Conclusion:**

Our results show that the expression of heterologous proteins coded by high RIL codon content coding sequences in a codon bias-adjusted strain is detrimental for their solubility. Our data support the hypothesis that the possible elimination of translational pauses that increase translation rate leads to protein misfolding and aggregation. This stresses the importance of strain selection according to codon content in any scheme where a large amount of biologically active product is desirable.

## Background

In research and industry, obtaining correctly folded recombinant proteins for downstream utilization is a major challenge. Many analysis techniques such as crystallography, nuclear magnetic resonance, circular dichroism and other emerging functional genomics approaches require considerable amounts of soluble protein. Likewise, commercial enzyme production has dramatically increased over the years. *Escherichia coli *is the system of choice for overexpressing heterologous proteins [1]. As a host, this bacterium has numerous advantages, including inexpensive culture conditions, very well known genetic background, easy manipulation and amenability to high density fermentation procedures [1-3]. Still, persistent hindrances to the use of this host are the low level of expression for some proteins and the formation of inactive insoluble aggregates. These problems can arise due to product toxicity, mRNA instability, lack of posttranslational modification, saturation of the folding machineries of the host cell and cofactors deficiency [4]. In addition, depletion of low-abundance tRNAs occurs if the foreign mRNA contains many codons that are rare in *E. coli*. This deficiency may lead to amino acid misincorporation and/or truncation of the polypeptide, thus affecting the heterologous protein expression levels and quality [5]. Strategies for solving codon usage bias such as codon optimization of the foreign coding sequence by silent mutagenesis or increasing the availability of underrepresented tRNAs by host modification have been described [2,3,5]. The increases in expression levels achieved by both methods are remarkable [5]. While codon optimization is a cumbersome and expensive process, modifying host availability of rare tRNAs is an easier approach. This methodology led to the commercialization of bacterial strains carrying plasmids containing extra copies of problematic tRNAs genes.

Accumulated evidence indicates that modulation of the translational speed facilitates protein folding events [6]. Translation occurs at a discontinuous pace partly due to the distribution of synonymous codons. The presence of rare codons along the mRNA causes ribosome stalling allowing the newly synthesized chain to adopt a well-folded intermediate conformation. In some cases the substitution of rare codons by frequent ones resulted in protein misfolding and the loss of biological activity [7].

Although expression levels of heterologous proteins in *E. coli *have been improved by codon optimization and tRNA level augmentation [5], systematic analyses of the impact of these strategies on protein solubility are needed. Since protein folding is modulated by translational speed, an effect on the amount of soluble recombinant protein obtained in codon bias-adjusted strains is expected.

The aim of this work is to analyze the expression of a set of plant proteins in the *E. coli *BL21(DE3)-pLysS strain (BL) commonly used for protein expression and a codon bias-adjusted strain, the BL21(DE3)-CodonPlus-pRIL strain (CP). In the latter, tRNAs levels for the codons AGA/AGG_Arg_, AUA_Ile _and CUA_Leu_, (named from now on "RIL codons") have been augmented (Stratagene Newsletter, 14.2, p. 5053). Our results show that proteins coded by coding sequences having more than 5% of RIL codons (H-RIL proteins) were mainly insoluble when expressed in the CP strain. In contrast, when expressed in an unmodified BL strain, these proteins were more soluble. Moreover, the expression of proteins from high RIL codon content RNAs caused retarded growth and low cell yield in the CP strain regardless of the temperature of induction. On the contrary, proteins having less than 5% of amino acids coded by RIL codons (L-RIL proteins) were highly soluble in both strains and did not affect bacterial growth. Our results indicate that the expression of high RIL codon content coding sequences in a codon bias-adjusted strain is detrimental for protein solubility.

## Results and discussion

### Classification of plant proteins by their codon content frequencies

The frequencies of the 61 sense codons for the 20 amino acids as they occur in the mRNAs of eight plant coding sequences were calculated (Table 1). The two arginine codons AGA and AGG, the isoleucine codon ATA and the leucine codon CTA, which are frequent in plants (see Additional File 1), can be regarded as rare in *E. coli *because they occur at a frequency below 10 per 1000 codons [8,9]. We also calculated the number of total rare codons (TRC, Table 1) which includes the aforementioned four codons and all other rare codons. Then, the frequencies of RIL codons and TRC were computed as a percentage of the total number of codons in each coding sequence (Table 1). No specific patterns in the distribution of RIL codons or evident clusters were found (Additional File 2). The proteins used in this study were sorted in two groups as following: the H-RIL group encompasses coding sequences having more than 5% of RIL codons (arbitrary cut-off value) while the L-RIL group includes coding sequences containing less than 5% of RIL codons.

**Table 1:** Rare codon frequencies of the coding sequences used in this study

Coding sequence	Total codons	RIL codon content^a^			% of RIL codons	% of Rare codons^b^	GC Content (%)	Description^c^
						
		Arg AGA/AGG	Ile ATA	Leu CTA				
*fd*	97	1/1	0/4	1/6	2.1	24.7	40	pea ferredoxin
*clpt1*	178	3/4	1/8	2/20	3.4	9.6	48	05-08-O17 – accessory protein of the chloroplastic ClpP protease complex
*fnr*	308	7/8	2/12	2/21	3.6	19.8	42	pea ferredoxin-NADP(H) reductase
*clpp4*	235	6/11	4/26	0/18	4.3	20.4	44	04-15-O12 – component of the chloroplastic ClpP protease complex
*clpc2*	834	32/64	10/61	9/82	6.1	12.6	46	09-19-G11 – chloroplastic chaperone
*clpd*	865	33/56	16/62	8/85	6.4	15.1	46	05-05-I08 – chloroplastic chaperone
*clpr2*	224	10/18	4/13	4/19	8.0	21.9	47	06-10-N03 – component of the chloroplastic ClpP protease complex
*dsRBD2*	82	6/6	0/2	7/19	8.5	23.2	43	Second double-stranded RNA-binding domain of DICER1 (*Arabidopsis thaliana*)
*trx*	109	0/1	0/9	0/13	0.0	3.7	52	*E. coli *thioredoxin
*E. coli*	-	-	-	-	-	-	51	*Escherichia coli *K12 NCBI RefSeq Accession NC000913
*A. thaliana*	-	-	-	-	-	-	36^d^	Whole genome data at NCBI

^a^Ratio of RIL codon(s) for the indicated amino acid to the number of total codons encoding each amino acid, respectively. RIL codons are indicated in parenthesis below each amino acid.

^b^Percentage of codons previously defined as rare in *E. coli *because they occur at frequency below 10% [8,9]

^c^Numbers indicate clone number from the RIKEN Arabidopsis full length cDNA bank. All clp coding sequences are from *A. thaliana*

^d^All genome data with the exception of mitochondrial DNA.

### Effect of the expression of heterologous proteins from H-RIL and L-RIL coding sequences on the growth of *E. coli *host cells

**Figure 1:**
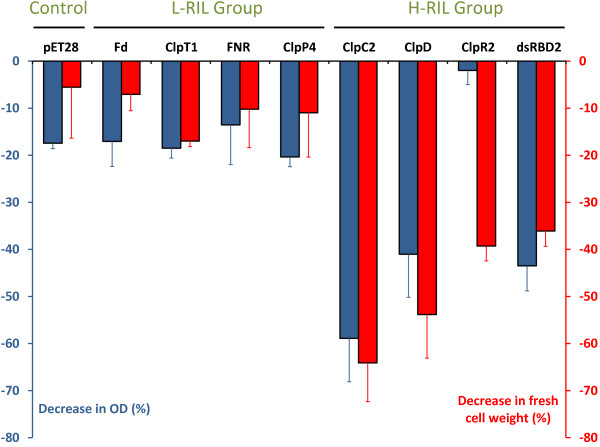
**Effect of protein expression on CP cells growth**. Cells carrying each plasmid were grown at 25°C for 6 h as described in Methods. Blue bars show the percentual change in final OD_600 _while red bars show percentual change in fresh cell weight of the induced culture vs the uninduced culture. Each bar represents the mean (plus error bars) of three independent experiments.

The overexpression of heterologous proteins in *E. coli *imposes a metabolic stress on the host strain that may lead to reduced cell growth and decreased yield of the target protein. Diverse effects on *E. coli *growth have been observed during overexpression of endogenous [10] or heterologous proteins [11]. To investigate the effect of the expression of the different plant proteins on *E. coli *growth, the recombinant cells were grown to mid-exponential phase up to an optical density at 600 nm of about 0.5 in LB medium containing the appropriate antibiotics. Then, expression of recombinant proteins was induced by the addition of 0.5 mM IPTG at 25°C for 6 h. After this time lapse, the OD_600 _and fresh cell weight were determined. As shown in Figure 1, expression of H-RIL proteins in the CP strain produced growth retardation in all cases compared to the normal growth of the strain harboring the corresponding plasmid but without induction. The effect was noticeable not only on the final OD_600 _of the cultures but also in the final cell wet weight to a similar extent (Figure 1) with the exception of the strain expressing ClpR2. In this case, the decrease in fresh cell weight was much higher that the decrease in OD_600_. A possible explanation for this result is that expression of ClpR2 caused morphological changes in the *E. coli *cells which acquire a round shape. These changes are different from those observed for cells expressing the other proteins (Additional File 3). On the other hand, the BL strain growth was less affected by the expression of H-RIL proteins. Quite the opposite, expression of L-RIL proteins produced only minor effects on both strains (Figure 1 and Additional File 4). Interestingly, growth retardation correlated with the frequency of RIL codons but not with the frequency of TRC of the expressed protein. For example, the pea ferredoxin coding sequence has the highest frequency of TRC but the lowest content of RIL codons (Table 1), yet its expression did not cause a negative effect on cell growth. In the same way, no correlation between CG content of the expressed coding sequence and bacterial growth was found (Figure 1 and Table 1). To examine if growth arrest was a consequence of cell lysis during protein expression, protein accumulation in the culture medium was analyzed as previously described [12]. No evidence of cell lysis was found for any of the expression conditions used (not shown). When cells from both strains bearing the empty vector were used as controls under identical conditions, a decrease of cell growth of about 10% (BL strain) and about 17% (CP strain) was observed.

It has been described that overexpression of a phage lambda protein containing rare codons in a wild type *E. coli *strain inhibited cell growth and protein synthesis [11]. This effect was overcome by changing the rare codons in the expressed gene or by supplementing the strain with appropriate tRNAs [13]. However, in our case the growth inhibition observed during expression of H-RIL proteins in the CP strain cannot be attributed to a deficiency in specific RIL-tRNAs. The effect may be related to the consumption of other limiting molecules (tRNAs, amino acids) or by energy depletion as a result of the increase in protein synthesis.

It has been proven that inclusion body formation results from an unbalanced equilibrium between protein aggregation and solubilization [14,15]. As suggested by these authors, when protein synthesis is carried out at high rates the system responsible for protein disaggregation in the cell may be saturated. The participation of molecular chaperones in the solubilization of protein aggregates has been well established [16]. Expression of the VP1 capsid protein of foot-and-mouth disease virus in *E. coli *resulted in the production of inclusion bodies. In this case, it was observed that these inclusion bodies did not produce a detectable toxicity to the bacterial cells. However, in strains deficient in the main chaperones DnaK or GroEL, the expression of this foreign protein caused a dramatic reduction of cell viability. [17]. It has been suggested by these authors that an increase in the inclusion body surface would be a key determinant of toxicity. If the increase in translational rate results in massive inclusion body formation, then this could overwhelm the chaperone system, ultimately causing cytotoxicity.

### Expression levels and solubility of the different plant proteins in *E. coli*

**Figure 2:**
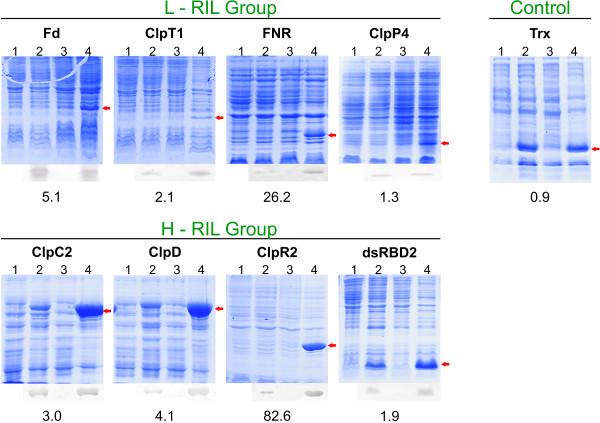
**Protein expression in whole lysates**. SDS-PAGE and Western blots of total cell proteins. BL uninduced and induced cultures: lane 1 and 2. CP uninduced and induced cultures: lane 3 and 4. Each coding sequence product is marked with a red arrow. Numbers below each blot indicate the fold increase in protein expression in the CP strain compared with the BL strain. The polyacrylamide percentage of each gel was as follow: 10% ClpC2 and ClpD, 12% FNR, ClpP4 and ClpR2 and 15% Fd, ClpT1, dsRBD2 and Trx. To fulfil linearity and detection limits for the inmunodetection method, a fraction of sample loaded in Coomassie Blue stained gels were loaded in Western blots as follows: Fd and FNR (lane 4), 20%; ClpC2, ClpD and dsRBD2 (lane 2), 20%; ClpC2, ClpD, ClpR2 and dsRBD2 (lane 4), 10%.

To determine the levels of expression and the amount of soluble protein, induction of expression of all proteins under study was performed in both strains using identical conditions as described in Methods and compared to uninduced *E. coli *cells carrying the expression vectors. After cultivation, cells were harvested by centrifugation, resuspended in cold lysis buffer to an OD_600 _~20 and disrupted by sonication. Then, identical amounts of whole lysates were analyzed by SDS-PAGE and Western blotting. Bands with the expected molecular masses were observed for each expressed protein in whole samples (Figure 2) and the amount of each expressed protein was determined using densitometric analysis of the blots. Numbers below each electrophoresis gel indicate the relative change observed for the expression of each protein in the codon bias-adjusted strain with respect to the BL strain. In all cases, protein expression was improved using the CP strain. There are many reports showing that protein production can be enhanced significantly by co-expressing the cognate tRNA coding sequences compared to the expression levels of conventional BL21(DE3) cells [18-20].

**Figure 3:**
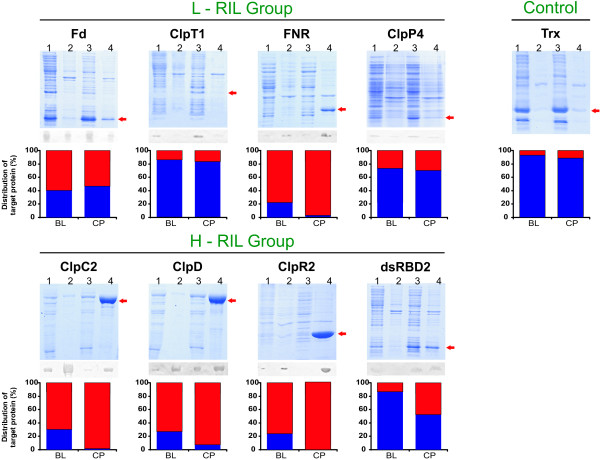
**Distribution of each protein in the soluble and insoluble fractions**. SDS-PAGE and Western blots of soluble and insoluble fractions from induced BL (lanes 1 and 2) and CP (lane 3 and 4) cultures. Lane 1 and 3 are supernatants obtained by centrifugation of whole lysates at 10,000 g for 30 min. Lane 2 and 4 are the obtained pellets resuspended with the same volume of buffer as the supernatants. Bar plots show the percentual amount of target protein in each fraction for both strains (blue: supernatant, red: pellet). To fulfil linearity and detection limits for the inmunodetection method, a fraction of sample loaded in Coomassie Blue stained gels were loaded in Western blots as follows: FNR (lane 4), 20%; ClpC2, ClpD and ClpR2 (lane 4), 10%.

Next, the propensity of each overexpressed protein to be soluble was investigated. Accordingly, the lysates were centrifuged at 10,000 g for 30 min under cold conditions and the same amount of total protein from each supernatant was analyzed by SDS-PAGE and Western blotting (Figure 3, lanes 1 and 3). Pellets were resuspended in the same amount of buffer as the supernatants and were loaded onto the electrophoresis gels (Figure 3, lanes 2 and 4). Below each blot the percentage of protein which was found in the pellet (red) or in the supernatant (blue) is presented. A decrease in the percentage of soluble protein was observed for H-RIL proteins expressed in the codon bias-adjusted strain. In contrast, L-RIL proteins and the bacterial thioredoxin used as a control displayed similar high solubility in both strains. It is worth to mention that thioredoxin has no amino acids coded by RIL codons (Table 1).

As previously suggested, codon bias seems to be relevant for the production of target proteins since it acts on the translation rate, though many other factors have been suggested to influence the process. It has been recently proposed that long-enough ribosomal pause time scales may lead to alternate folding pathways [21]. During synthesis and protein folding different folding pathways leading to trapped states may impede obtaining properly folded molecules. These trapped states are more likely to be formed if protein synthesis and folding occurs simultaneously; facilitating unfavourable interactions between different domains [21]. Thus, some infrequent codons may introduce long-enough ribosomal pauses to allow the nascent protein to fold sequentially. This may lead to different folding pathways reaching distinct minima, and subsequently increasing the amount of properly folded protein. Recently, Zhang *et al*. [22] using a bioinformatics approach identified putative sites of translational attenuation by codon selection in about 60% of the total *E. coli *ORFs. The authors provide evidences that discontinuous elongation of a peptide chain due to slow-translating clusters may be particularly important for protein folding. This phenomenon may have profound effects on protein folding and may explain in part our experimental observations. It has been reported that co-overexpression of the cognate *argU *tRNA during production of the yeast α-glucosidase in *E. coli *increases translation rate but stimulates aggregation [23]. In this particular case the total active protein, which is rich in AGA and AGG codons, was about one third less when expressed in a codon bias-adjusted strain with respect to the parental host, albeit the total expressed protein was increased four times [23].

**Figure 4:**
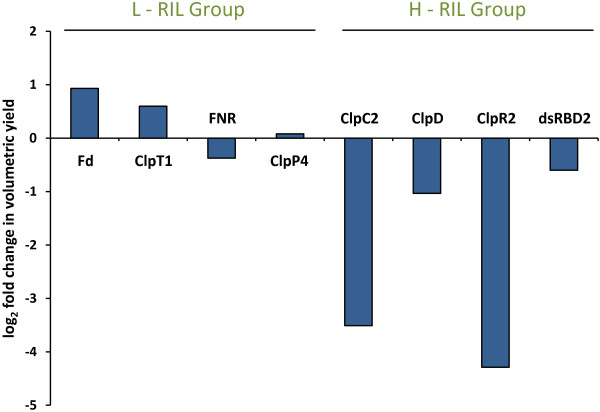
**Fold change in soluble protein volumetric yield**. Fold change represents the log_2 _of the ratio of the volumetric yield of each protein (in mg per liter of soluble protein) expressed in the CP strain and the volumetric yield of the same protein expressed in the BL strain. Protein expressed at a constant level (ratio of 1) has a log2(ratio) equal to zero, which can be seen as "no change".

The yield of the expressed proteins per liter of culture was calculated and it is shown in Figure 4. Since the expression of H-RIL proteins affects bacterial growth and protein solubility in the CP strain, the total amount of H-RIL proteins in soluble form per liter of *E. coli *culture is significantly reduced when compared with the yield of the same proteins in the BL strain. It cannot be ruled out that the expressivity of the analyzed coding sequences correlates with bacterial growth rate. In this case, proteins that are expressed to higher levels may slow down bacterial growth rate, regardless of their solubility. However, the high level expression of glutathione S-transferase as a soluble protein without affecting bacterial growth (not shown) suggests that, for the set of protein tested, accumulation of inclusion bodies are probably the cause of the observed decrease in cell viability.

A common strategy for achieving better protein solubility is lowering the temperature upon induction. By doing this, the overall rate of protein synthesis is lowered, thus preventing recombinant proteins from saturating the cellular folding machinery. Accordingly, we tested whether lower temperatures could be an effective way of keeping aggregation-prone proteins of the H-RIL group in a soluble form. When expression was performed at 17°C, the partition of all proteins in the soluble and insoluble fraction was relatively the same as previously observed at 25°C in both strains (not shown). It appears that even by lowering the temperature, the rate of translation in the CP strain is still high enough to prevent H-RIL proteins to fold properly.

**Figure 5:**
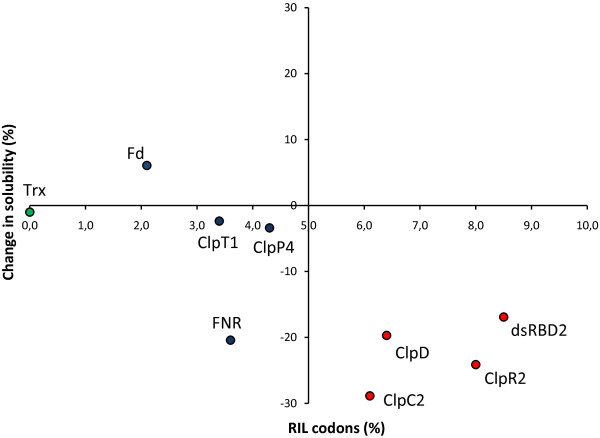
**Change in solubility of target proteins as a function of RIL codon content**. Change in solubility indicates the difference of the percentage of soluble protein in the CP strain with respect to the percentage of soluble protein in the BL strain. Blue dots, L-RIL proteins; red dots, H-RIL proteins; green dot, thioredoxin.

To facilitate the analysis, the obtained data is presented in Figure 5 which represents the detected change in solubility for each protein as a function of the percentage of RIL codon content. Interestingly, the two groups are clearly discriminated with the exception of FNR. This protein contains the prosthetic group FAD [24] and its incorporation may influence its proper folding and solubility.

## Conclusion

Rare codons are thought to facilitate protein folding by slowing down RNA translation at specific sites. In this work it is shown that some proteins containing RIL codons become more insoluble when overexpressed in a codon bias-adjusted *E. coli *strain. In this strain, the translational pauses introduced by the RIL codons are probably overridden, increasing translation speed and consequently, protein aggregation. Moreover, coding sequences with high RIL codon content produced growth retardation when expressed in BL21(DE3)-CodonPlus-pRIL. At all temperatures tested, a decrease in the percentage of soluble protein was observed for H-RIL coding sequences expressed in the codon bias-adjusted strain. The combined effect of bacterial growth retardation and decrease in protein solubility significantly reduced the total amount of soluble protein obtained per liter of *E. coli *culture.

In conclusion, by analyzing the RIL codon content of the coding sequence to be expressed, a proper bacterial host can be chosen so as to improve the production of properly folded proteins.

## Methods

### Strains and plasmids

For protein expression *E. coli *strains BL21(DE3)-pLysS (Novagen) and BL21(DE3)-CodonPlus-pRIL (Stratagene) were used. The latter carries extra copies of the coding sequences encoding the *argU*, *ileY*, and *leuW *tRNAs, for codons AGG/AGA, AUA, and CUA respectively.

The plant coding sequences used throughout this study were expressed under the control of the T7 promoter using vector pET28a from Invitrogen. All expression constructs encoded an N- or C-terminal His_6 _tag. Chloroplast proteins were expressed as their mature forms (i.e., without the transit peptide).

The vectors carrying the *fnr *and *fd *coding sequences from pea were described previously [25,26]. The Trx containing plasmid is pET32 from Stratagene. The cDNA for all other coding sequences were obtained from the RIKEN cDNA bank [27]. The cDNAs for *clpc2*, *clpd *and *dsRBD2 *were amplified by PCR and cloned between the NheI and EcoRI (*clpc2 *and *dsRBD2*) and NheI and NotI (*clpd*) restriction sites. *clpt1*, *clpr2 *and *clpp4 *were cloned in a modified version of pET28a which tags the proteins at their C-teminal end. Briefly, pET28a was digested with NcoI and XhoI, in this way the N-terminal His-tag sequence and the thrombin cleavage site were eliminated. Then, *clpt1 *was amplified with an upper primer containing a NcoI site and a lower primer that contained an EcoRI site, the His-tag sequence, the thrombin cleavage site and a XhoI restriction site *in tandem*. The PCR product was cloned in pET28a producing pClpT1. This vector was used to clone *clpr2 *and *clpp4 *in the NcoI and EcoRI restriction sites.

All constructions were checked by DNA sequencing and transformed into the BL or CP strains.

### Culture conditions

For expression studies, chemically competent BL or CP cells were transformed with 5 ng plasmid and grown overnight on agar plates with appropriate antibiotics. A 2 ml preculture in LB medium was started from an isolated colony and grown overnight at 37°C. The day after, the OD_600 _was measured in an Ultrospec 110 spectrophotometer (Amersham Biosciences) and 100 ml bottles containing 10 ml of LB medium with antibiotics were inoculated at a final OD_600 _of 0.05 with the preculture. The culture was grown at 37°C for 2 h up to OD_600 _≈ 0.5. Then, the temperature was lowered to 25°C or 17°C and the heterologous protein expression induced by addition of 0.5 mM IPTG. For pea ferredoxin, a mixture of Fe^2+^-EDTA (0.024% p/v and 0.06 mM respectively) was also added along with the inducer. Non-induced cultures were used as controls. After 6 h (25°C) or 18 h (17°C) of continuous growth, the final OD_600 _was recorded. For fresh cell weight determination, cells were collected in pre-weighted 15 ml conical tubes and harvested by centrifugation. Then, the supernatant was discarded and the weight determined using an analytical balance. This procedure was repeated twice in order to eliminate all traces of liquid. Cell pellets were stored at -70°C until protein analysis.

### Protein analysis

Cell density for each culture was normalized to an OD_600 _of 20 into lysis buffer (50 mM Tris HCl pH 7.5, 150 mM NaCl, 1 mM phenylmethylsulphonyl fluoride). The cell suspension was disrupted by sonication for 5 min on ice until complete cell lysis was achieved. An aliquot (10 μl) of total lysates were used to analyze total protein expression by SDS-PAGE. In parallel, lysates were subjected to centrifugation (10,000 g, 30 min, 4°C), the supernatants separated and analyzed for protein concentration by a standard procedure [28]. Supernatant samples containing 20 μg of total protein were analyzed by SDS-PAGE. Pellets were resuspended in the same amount of buffer as the supernatants and equivalent volumes loaded onto the electrophoresis gels. All gels contained a molecular weight marker (Full Range Rainbow Molecular Marker, GE Healthcare, USA) and BSA as an internal standard. Different percentages of polyacrylamide gels were used depending on the molecular weight of the protein of interest as stated in each case. Samples were also analyzed by Western blotting. Immunodetections were carried out using a His-Probe antibody (Santa Cruz Biotechnology, CA) followed by an anti-rabbit alkaline phosphatase-conjugated antibody (GE Healthcare, USA). Detection with the chromogenic substrate 5-bromo-4-chloro-3-indolyl phosphate/nitro blue tetrazolium chloride was carried out as recommended by the suppliers. Quantification was performed by scanning the blots after immunodetection and subsequent analysis of the data with GelPro Analyzer software (Media Cybernetics, Silver Spring, MA).

## Abbreviations

BL: BL21(DE3)-pLysS; Clp: caseinolytic protease; CP: BL21(DE3)-CodonPlus-pRIL; dsRBD2: double-stranded RNA-binding domain; H-RIL protein: a protein having more than 5% of amino acids coded by RIL codons; IPTG: Isopropyl-D-galactoside; LB: Luria-Bertani; L-RIL protein: a protein containing less than 5% of amino acids coded by RIL codons; OD_600_: Optical density at 600 nm; SDS-PAGE: Sodium dodecyl sulfate-polyacrylamide gel electrophoresis; TRC: total rare codons.

## Competing interests

The authors declare that they have no competing interests.

## Authors' contributions

GLR carried out the experiments and drafted the manuscript. EAC supervised the experiments and drafted the manuscript. Both authors read and approved the final manuscript.

## Supplementary Material

Additional file 1 **Rare codon frequencies in *Escherichia coli *and model photosynthetic organisms**. Codon frequencies (per 1000) extracted from the Kazusa codon usage database . Click here for file

Additional file 2 **Distribution of rare codons in the coding sequences under study**. Blue bars represent the frequency (per 1000) of a particular codon in each studied coding sequence. RIL codons are represented as red bars. A yellow dotted line indicates a codon frequency of 10 × 1000. Frequencies are those for *E. coli *and were taken from the Kazusa codon usage database . Click here for file

Additional file 3 **Effect of protein expression on bacterial morphology**. Representative light micrographs of *E. coli *cultures. A) Cells overexpressing ClpP4 (L-RIL). B) Cells overexpressing ClpC2 (H-RIL). C) Uninduced and D) induced cells bearing the ClpR2 expression vector. Cells were observed before induction with IPTG (C) or 6 h after induction at 25°C (A, B and D). Click here for file

Additional file 4 **Effect of protein expression on BL growth**. Blue bars show the percentual change in final OD_600 _while red bars show percentual change in fresh cell weight of the induced culture vs the uninduced culture. BL cells carrying each plasmid were grown at 25°C for 6 h. Each bar represents the mean (plus error bars) of three independent experiments. Click here for file
