# Supplementary material for: Rare codon content affects the solubility of recombinant proteins in a codon bias-adjusted Escherichia coli strain
Source: Microb Cell Fact. 2009 Jul 24;8:41. doi: 10.1186/1475-2859-8-41 (PMC2723077; doi:10.1186/1475-2859-8-41)
Supplement: Additional file 1 — Rare codon frequencies in Escherichia coli and model photosynthetic organisms. Codon frequencies (per 1000) extracted from the Kazusa codon usage database . [file 1475-2859-8-41-S1.pdf]

Additional Table 1

Rare codon frequencies in *Escherichia coli* and model photosynthetic organisms

| | Codon frequency | | | | | | |
|---|---|---|---|---|---|---|---|
| | Arg (*arg* U) | | Ile (*ile* Y) | Leu (*leu* W) | Pro (*pro* L) | Ser | Thr |
| | AGA | AGG | AUA | CUA | CCC | UCA | ACA |
| *Escherichia coli* | 2.4 (5.10) | 2.1 (4.12) | 5.0 (7.15) | 3.4 (2.81) | 2.4 (5.93) | 6.1 (10.65) | 6.1 (11.38) |
| **Protists** | | | | | | | |
| *Chlamydomonas reinhardtii* | 0.7 (1.24) | 2.7 (4.79) | 1.1 (3.08) | 2.6 (2.90) | 29.5 (46.53) | 3.2 (4.89) | 4.1 (7.75) |
| *Synechococcus elongatus PCC 7942* | 1.6 (2.36) | 1.0 (1.48) | 1.0 (1.84) | 11.2 (9.13) | 18.3 (33.52) | 5.6 (9.33) | 7.2 (13.85) |
| **Plants** | | | | | | | |
| *Arabidopsis thaliana* | 19.0 (35.19) | 11.0 (20.37) | 12.6 (23.95) | 9.9 (10.59) | 5.3 (10.88) | 18.3 (20.49) | 15.7 (30.66) |
| *Pisum sativum* | 16.6 (35.24) | 11.0 (23.35) | 13.3 (23.92) | 8.8 (9.85) | 6.3 (12.40) | 17.9 (22.80) | 17.5 (31.99) |
| *Nicotiana tabacum* | 16.0 (32.92) | 12.2 (25.10) | 14.0 (25.13) | 9.4 (10.26) | 6.6 (13.17) | 17.6 (23.04) | 17.4 (33.53) |
| *Solanum lycopersicum* | 16.4 (35.04) | 11.9 (25.43) | 14.0 (24.91) | 10.0 (10.50) | 5.7 (11.70) | 20.7 (25.27) | 17.9 (35.10) |
| *Lemna gibba* | 15.1 (25.21) | 11.8 (19.70) | 4.7 (13.39) | 3.2 (4.42) | 25.5 (35.32) | 9.2 (9.24) | 5.4 (12.05) |
| *Medicago truncatula* | 17.7 (35.40) | 12.1 (24.20) | 15.5 (26.45) | 10.2 (10.54) | 5.5 (12.22) | 21.2 (25.00) | 17.9 (35.51) |
| *Zea mays* L. | 8.8 (15.22) | 14.8 (26.61) | 8.4 (18.71) | 7.3 (7.86) | 13.5 (24.24) | 11.0 (14.80) | 10.5 (21.52) |
| *Oryza sativa* | 10.5 (15.09) | 16.0 (22.99) | 8.8 (20.76) | 7.7 (8.51) | 12.1 (20.90) | 12.4 (15.80) | 11.6 (23.92) |

Codon frequencies (per 1000) were extracted from the Kazusa codon usage database (http://www.kazusa.or.jp/codon/).
Values in parenthesis indicate the percentage of each specific codon among the codons for the amino acid stated.
