# Supplementary figures and images for: Rare codon content affects the solubility of recombinant proteins in a codon bias-adjusted Escherichia coli strain
Source: Microb Cell Fact. 2009 Jul 24;8:41. doi: 10.1186/1475-2859-8-41 (PMC2723077; doi:10.1186/1475-2859-8-41)

## L-RIL Group

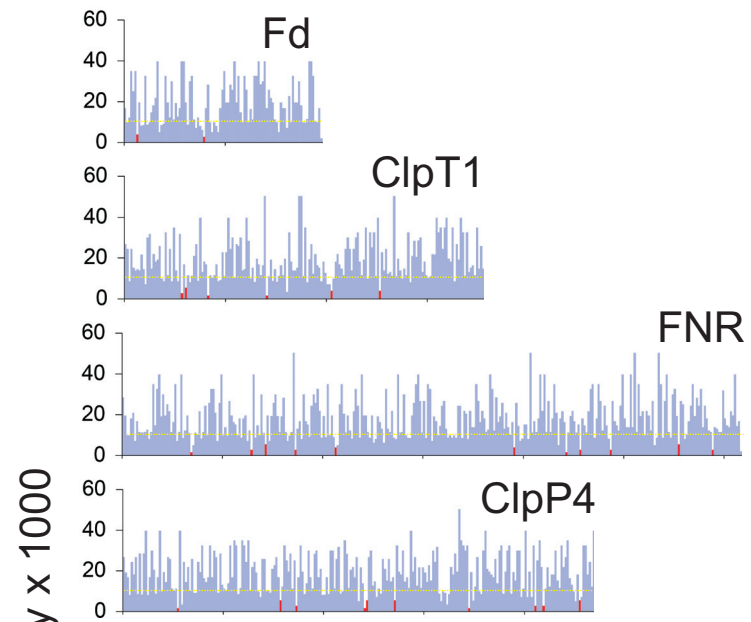

## H-RIL Group

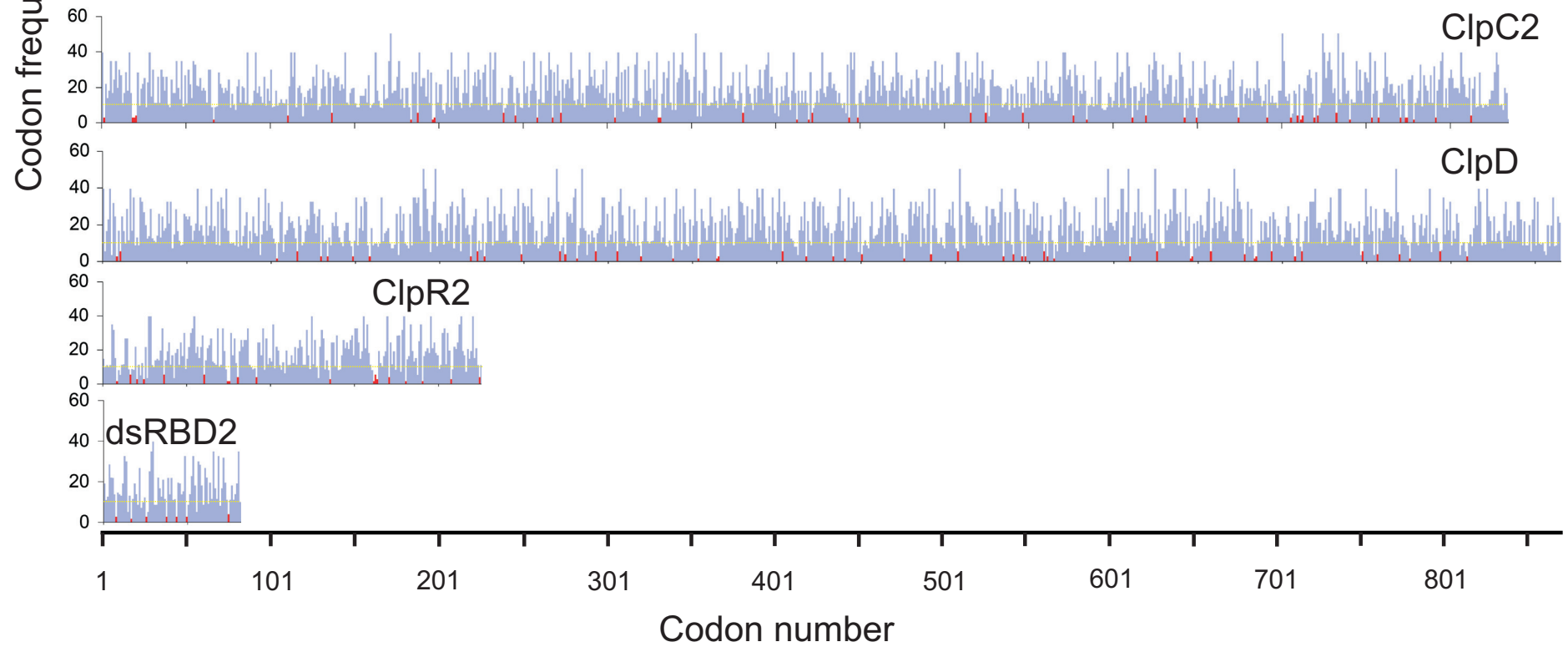

Supplement: Additional file 2 — Distribution of rare codons in the coding sequences under study. Blue bars represent the frequency (per 1000) of a particular codon in each studied coding sequence. RIL codons are represented as red bars. A yellow dotted line indicates a codon frequency of 10 × 1000. Frequencies are those for E. coli and were taken from the Kazusa codon usage database . [file 1475-2859-8-41-S2.pdf]

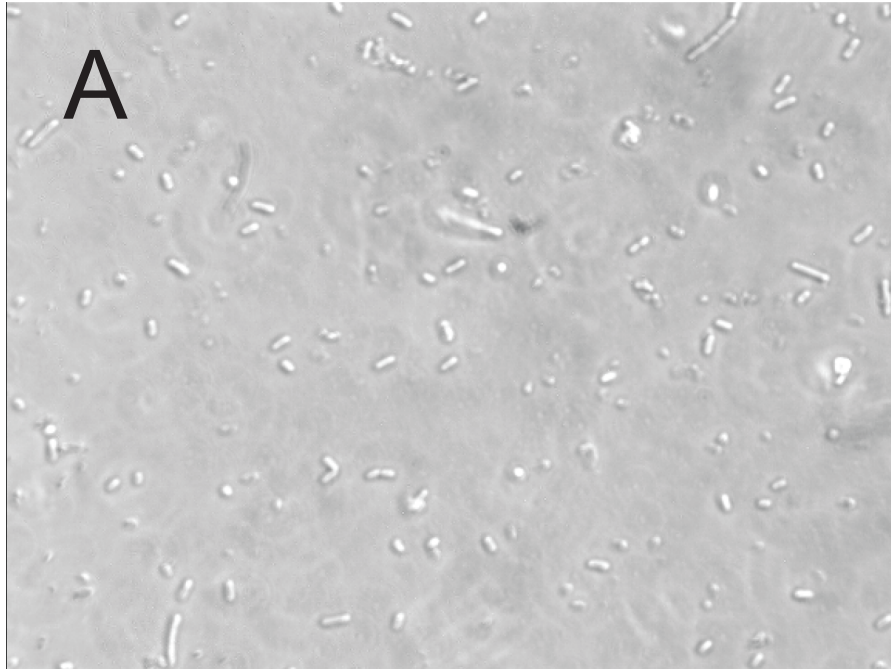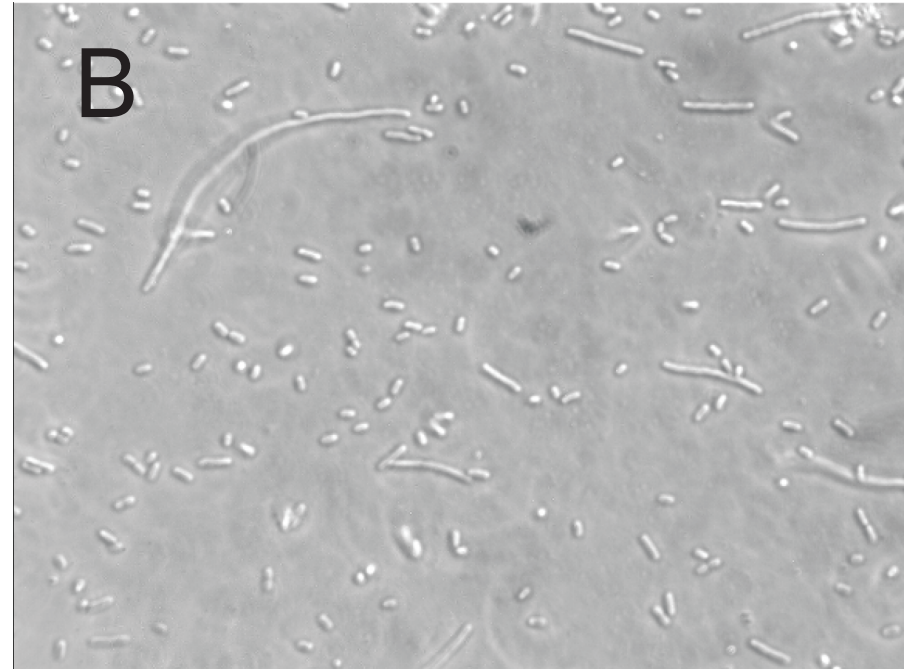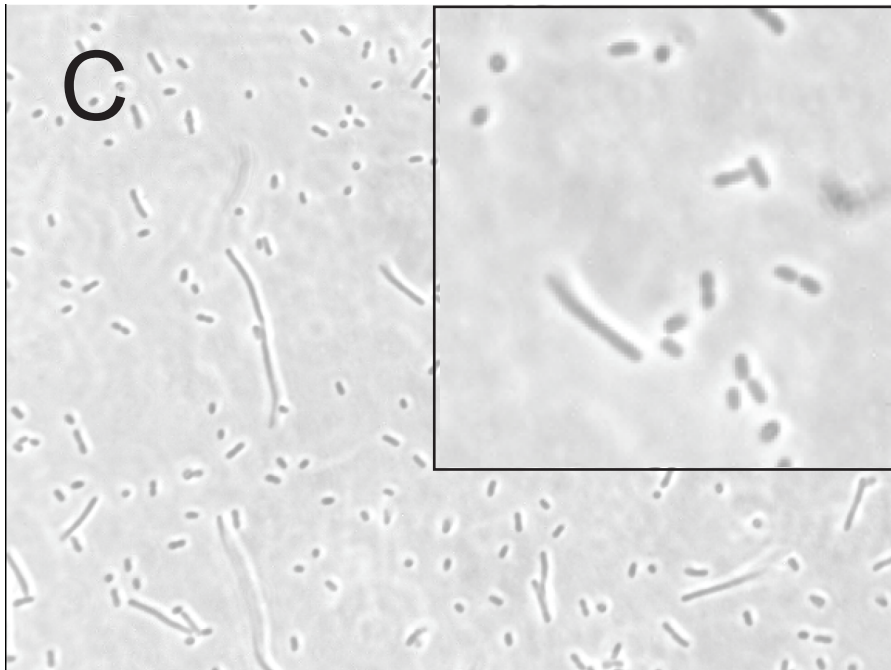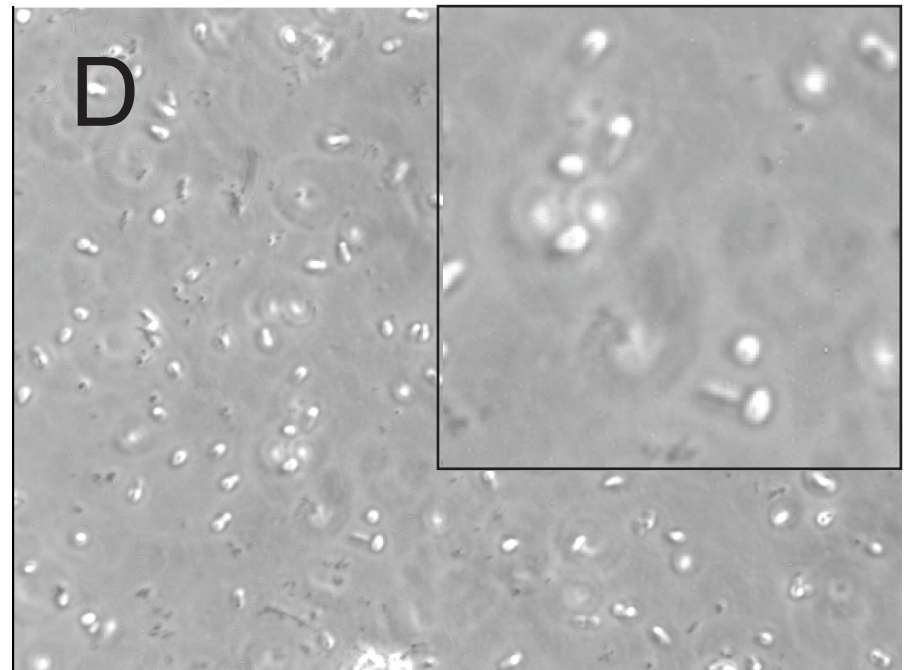

Supplement: Additional file 3 — Effect of protein expression on bacterial morphology. Representative light micrographs of E. coli cultures. A) Cells overexpressing ClpP4 (L-RIL). B) Cells overexpressing ClpC2 (H-RIL). C) Uninduced and D) induced cells bearing the ClpR2 expression vector. Cells were observed before induction with IPTG (C) or 6 h after induction at 25°C (A, B and D). [file 1475-2859-8-41-S3.pdf]

Control

L-RIL Group

H-RIL Group

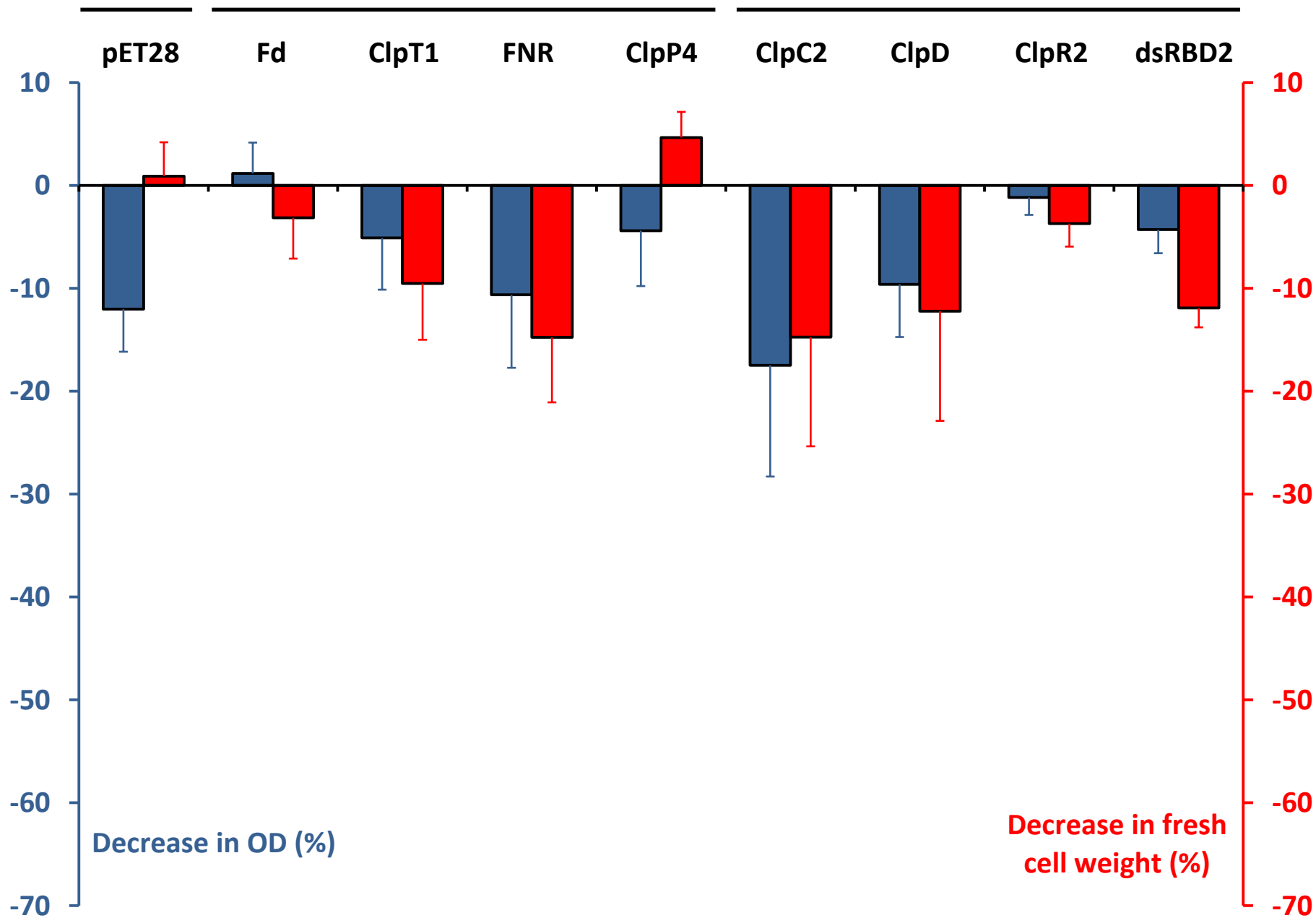

Supplement: Additional file 4 — Effect of protein expression on BL growth. Blue bars show the percentual change in final OD600 while red bars show percentual change in fresh cell weight of the induced culture vs the uninduced culture. BL cells carrying each plasmid were grown at 25°C for 6 h. Each bar represents the mean (plus error bars) of three independent experiments. [file 1475-2859-8-41-S4.pdf]
